# From physical-mental synergy to health action: the mechanism path of promoting physical activity based on the interaction of cognitive body assessment and affective body assessment

**DOI:** 10.3389/fpsyg.2026.1880694

**Published:** 2026-07-23

**Authors:** Jiyan Xu, Jinghui Zhong, Bawuerjiang Mulatebieke, Chang Lu

**Affiliations:** 1 School of Sports Management and Communication, Capital University of Physical Education and Sports, Beijing, China; 2 School of Kinesiology and Health, Capital University of Physical Education and Sports, Beijing, China; 3 School of English Studies, Beijing Foreign Studies University, Beijing, China; 4 Department of Physical Education, Beijing University of Chinese Medicine, Beijing, China

**Keywords:** affective body assessment, cognitive body assessment, exercise motivation, knowing–doing gap, physical activity

## Abstract

**Objective:**

To address the issue of insufficient physical activity and the knowing-doing gap in exercise participation among college students, this study integrates the multidimensional connotations of body image to clarify the effects and mechanisms of cognitive body assessment and affective body assessment on college students’ physical activity, thereby providing support for addressing students’ exercise dilemmas and facilitating the adoption of healthy behaviors.

**Methods:**

A survey was conducted among 2,016 college students using the Multidimensional Body-Self Relations Questionnaire, the Sport Motivation Scale, and the Physical Activity Rating Scale. Statistical analyses, including correlation and regression analyses, were performed using SPSS 26.0, MATLAB, and Stata 16.0. A targeted model was developed and its goodness of fit was examined.

**Results:**

(1) No sex differences were found in cognitive body assessment or affective body assessment; however, sex differences were observed in exercise motivation and physical activity, with males exhibiting greater sensitivity to the cognition–affect combination and females showing a more pronounced effect of cognition. (2) Cognitive body assessment and affective body assessment exerted synergistic influences on college students’ physical activity, positive affect compensated for low cognitive levels in compensating for cognitive deficits, whereas solely enhancing affect showed no evident effect at high cognitive levels. (3) Exercise motivation mediated the effect of cognitive and affective body assessments on physical activity. The mediating effect of intrinsic motivation was significantly stronger than that of extrinsic motivation. Affective evaluation amplified the indirect effect of cognitive evaluation on physical activity through motivation.

**Conclusion:**

A comprehensive approach integrating the dual-track education of cognition and affect, optimization of campus exercise environments, precise intervention, and the construction of long-term mechanisms should be implemented to promote college students’ participation in physical activity and cultivate sustainable exercise habits.

## 1 Introduction

For the past two decades, the prevalence of physical inactivity has remained persistently high in most countries worldwide, with approximately 80% of adolescents and one-third of adults failing to meet the physical activity guidelines set by the World Health Organization (Ramirez et al., 2026; WHO, 2022). Research has indicated that physical activity among college students is significantly negatively correlated with negative emotions such as depression and anxiety (Xu et al., 2026), and that a longer duration of Moderate-to-Vigorous Physical Activity (MVPA) is associated with a lower risk of psychological symptoms (Deng et al., 2024). Currently, health problems among the college student population are particularly prominent, characterized by a significant decline in physical fitness and a marked increase in cardiovascular risk burden (Cai et al., 2025), which has become a global public health issue. The university stage, as a critical period of transition from adolescence to adulthood, represents a golden window for the formation and consolidation of healthy behaviors. Participation in exercise during this period not only affects students’ academic performance and mental health during their studies (Zhou and Lin, 2025) but also plays an important role in the long-term management of chronic diseases (Woelfel et al., 2024). However, exercise avoidance behaviors have become increasingly common among college students, and insufficient physical activity has become a widespread global problem, posing a serious health threat. Promoting healthy behaviors-particularly participation in physical activity-among college students has become an urgent practical issue that needs to be addressed.

In the field of health behavior promotion, the knowing–doing gap in exercise participation remains a core challenge. Over the past several decades, major frameworks for understanding physical activity have focused on the role of cognitive factors (Rhodes et al., 2019). Traditional cognitively oriented theories, such as the Health Belief Model and Protection Motivation Theory, place emphasis on how individuals reflect on their thoughts and feelings, overstating an individual’s ability and willingness to make rational decisions aimed at achieving desired goals (Ralf and Panteleimon, 2018). Although these theories have revealed the predictive role of health cognition in behavior, they have difficulty fully explaining why individuals often still fall into action dilemmas despite holding positive health cognitions. The limitations of these theories have gradually come to the forefront, as a marked discrepancy remains between people’s cognitions and behaviors-namely, the “knowing-doing gap.” Fábio contends that existing physical activity guidelines rely too heavily on epidemiological and medical evidence while neglecting the irrational characteristics of human behavior and the crucial role of affective experience-an oversight that may be a reason why global physical activity levels remain persistently low (Dominski et al., 2026). It has been suggested by a scholar that although many people rationally know that exercise is beneficial, they affectively experience it as unpleasant, which leads to an inability to persist in exercise. The fundamental reason is that individuals have not developed a good association between physical activity and positive affective attitudes (Ekkekakis and Zenko, 2016). Motivational hedonism holds that individuals tend to seek pleasant experiences and avoid unpleasant ones (Murphy and Eaves, 2016). However, most people associate exercise with unpleasant feelings (Ekkekakis and Zenko, 2016), which hinders their participation in physical activity. With the development of motor cognitive science and exercise psychology, dual-system theories and affective system theories that emphasize the role of affective factors have gradually gained prominence (Ekkekakis and Dafermos, 2012). Some scholars have found that exercise behavior is not merely the result of purely rational decision-making (Yan et al., 2022) but is rooted in bodily experience, interweaving automatic and controlled processes, and represents a complex adaptive performance deeply regulated by affective dynamics. In other words, the generation of exercise behavior is not a simple knowing-doing linear relationship but is jointly influenced by automatic affective evaluation and reflective cognitive processes (Ekkekakis and Brand, 2019), offering a new research perspective for addressing college students’ exercise participation dilemmas.

Body image is a multidimensional construct that encompasses an individual’s perceptions, attitudes, affect, and behaviors regarding physical appearance and functioning, and has since been extended to dimensions such as health functioning (Sabiston et al., 2025). The appreciation boundary defined by positive body image emphasizes the functionality and health of the body beyond mere appearance, whereas the respect boundary emphasizes the individual’s engagement in health-promoting exercise behaviors (Tylka and Wood-Barcalow, 2015). In contrast, body dissatisfaction, as a core manifestation of negative body image, is closely related to poor health, directly affects health-related behaviors, diminishes exercise motivation, and leads to exercise avoidance (More et al., 2019). Grounded in the multidimensional connotations of body image (Ma, 2006), this study defines cognitive body assessment as an individual’s rational recognition and objective judgment of their health status, and defines affective body assessment as an individual’s subjective affective response arising from the perception of their own health status. Accordingly, this study aims to examine the effects of cognitive body assessment and affective body assessment on college students’ physical activity, thereby providing theoretical and practical support for addressing students’ exercise participation dilemmas and facilitating the adoption of healthy behaviors.

## 2 Research hypotheses

Existing theories have revealed the driving factors of physical activity from various perspectives, yet two common gaps remain. First, the Affective-Reflective Theory (ART) and Self-Determination Theory (SDT) have primarily focused on individuals’ experiences and beliefs regarding the behavior of physical activity itself, paying less attention to how individuals’ global appraisals of their own physical and health status may serve as stable psychological resources that sustainably influence long-term physical activity participation. Second, although multiple theoretical frameworks have been cited in parallel, a unifying logical thread that integrates them into a coherent and testable hypothesis system is lacking.

To bridge these gaps, the present study proposes a theoretically integrative perspective grounded in the cognitive-affective processing system as its underlying logic, elevating the object of appraisal from specific exercise experiences to the meta-appraisal level of physical and health status. Specifically, this study extends the application boundary of ART from the immediate affective experience at the behavioral level to the affective disposition toward one’s own body at the self-perceptual level, and simultaneously refines the theoretical delineation of motivational sources within SDT by distinguishing between cognitively-driven and affectively-driven motivational pathways. At the interplay between cognition and affect, we further incorporate the Cognitive-Affective Consistency Theory, positing that the two types of appraisal not only exert independent effects but may also jointly moderate the efficiency of translating appraisals into physical activity through synergistic interaction. In operational terms, by integrating Behavioral Decision Theory, Affective–Reflective Theory (ART), Cognitive-Affective Consistency Theory, and the 24-h Cognitive-Affective-Body Behavior Model, this study examines Cognitive Body Appraisal (the rational pathway) and Affective Body Appraisal (the affective pathway) as dual antecedents, and their respective associations with exercise motivation and physical activity. Based on this integrated framework, specific hypotheses are proposed step by step as follows.

### 2.1 The relationship between cognitive body assessment and physical activity

Extant research generally holds that an individual’s cognitive judgment of their own health status exerts a significant influence on whether they choose to participate in physical activity and whether they can sustain it over the long term (Çiftci and Kadıoğlu, 2022). From the perspective of behavioral decision theory, when making decisions related to physical activity, individuals first draw on their own cognitive level to judge whether their health status needs to be improved through physical activity and whether engaging in physical activity can effectively enhance their health (Einhorn and Hogarth, 1981). This cognitive judgment serves as a core driving factor for individuals’ participation in physical activity. Further analysis drawing on self-efficacy theory reveals that an individual’s confidence in improving their health through physical activity encourages them to actively engage in physical activity, and this confidence is progressively strengthened through repeated participation in sports activities (Chalabaev and Sarrazin, 2019). Existing empirical studies also confirm the significant effect of individuals’ cognitive judgment of their own health status on their decisions to participate in physical activity and to sustain it over the long term. Chen et al. (2025) found that college students’ physical self-perception was significantly positively correlated with their level of physical activity, also emphasizing the role of self-perception in enhancing the positive effects of physical activity (Chen et al., 2025). In summary, positive perceptions of one’s own physical condition may be associated with higher levels of exercise participation. Therefore, this study proposes hypothesis:

*H1:* Cognitive body assessment is positively associated with physical activity.

### 2.2 The relationship between affective body assessment and physical activity

When individuals experience a sense of comfort and pleasure arising from their own health status, this can directly increase their level of physical activity participation. The Affective-Reflective Theory (ART) posits that individuals’ decisions about exercise behavior are not solely the result of conscious, deliberate cognitive reasoning but rather the outcome of competition and synergy between rapid, automatic affective responses and slow, cognition-based reflective evaluations (Ekkekakis and Dafermos, 2012). Conroy’s dual-system model of physical activity, which emphasizes the role of automatic affective evaluations, suggests that pleasant affective processes provide internal rewards that incentivize physical activity (Conroy and Berry, 2017). When individuals hold positive affective evaluations of their own health, they are more likely to generate and maintain exercise behaviors. That is, health perception-based affective body assessment constitutes an important psychological resource driving subsequent physical activity. Based on this, it can be further inferred that when this positive emotional response generalizes from the experience of specific physical actions to an overall evaluation of one’s own health status, individuals will also show a greater tendency to engage in physical activity. Therefore, this study proposes hypothesis:

*H2:* Affective body assessment is positively associated with physical activity.

### 2.3 The mediating role of exercise motivation in the effects of cognitive body assessment and affective body assessment on physical activity

From the cognitive pathway perspective, Wang et al. suggest that positive body perceptions, such as favorable cognitive appraisals of one’s body, can enhance individuals’ intrinsic motivation, prompting active engagement in physical activity (Shi et al., 2024). From the affective pathway perspective, research indicates that positive affective experiences are strong predictors of exercise motivation and subsequent physical activity behaviors (Furman et al., 2025). Positive health-related affective experiences promote physical activity by reinforcing exercise motivation, and the role of affect may even surpass that of traditional cognitive beliefs.

Saanijoki and Nummenmaa (2025) study uncovered the mechanisms through which affective and cognitive assessment influence exercise motivation: when individuals hold positive cognitive and affective appraisals of their own health status, interoceptive signals arising from exercise are more likely to be perceived as “vitality” or “positive incentives,” thereby reinforcing exercise motivation (Saanijoki and Nummenmaa, 2025). Williams (2008) drawing on hedonistic theory, proposed that individuals tend to repeat behaviors that elicit positive affective experiences. This affect-driven motivational process exists in parallel with cognitive decision-making processes, and the two may interact. When cognitive assessment and affective experiences are congruent, exercise motivation is maximally reinforced (Williams, 2008). Therefore, this study proposes hypothesis:

*H3:* Exercise motivation mediates the effects of both cognitive body assessment and affective body assessment on physical activity.

### 2.4 The moderating role of affective body assessment in the relationship between cognitive body assessment and physical activity

Cognitive-affective consistency theory suggests that individuals’ cognitive and affective processes do not operate independently but exert mutual effects and work synergistically, forming interaction effects that jointly influence behavioral choices. This interactive effect also exists in the domain of health and physical activity. Cognitive body assessment, as a rational health cognition, provides cognitive support for individuals’ participation in physical activity. Affective body assessment, as an affective health feeling, provides affective drive for individuals’ participation in physical activity. The synergistic effect of these two can more effectively promote physical activity participation. The 24-h Cognitive-Affective Physical Behavior Model explicitly states that there is a dynamic, bidirectional interaction between affective states and cognitive functions, in which affective responses not only independently influence physical behaviors but may also alter the direction and strength of the relationship between cognitive factors and behavior. The model emphasizes that affective assessment can shape individuals’ immediate experiences and subsequent judgments of physical activity, thereby potentially moderating the translation of rational cognition into actual behavior (Giurgiu and Priemer, 2025). This health perception-based affective assessment, which reflects an individual’ s overall emotional evaluation of their own health rather than a reaction to a specific activity, may more persistently influence the process of translating cognition into behavior. In summary, when individuals hold positive affective assessment of their own health status, the positive predictive effect of cognitive body assessment on physical activity may be enhanced. Therefore, this study proposes hypothesis:

*H4:* Affective body assessment moderates the relationship between cognitive body assessment and physical activity.

## 3 Materials and methods

### 3.1 Participants

Using random sampling, a questionnaire survey was conducted from early November to late December 2025. A total of 2,089 questionnaires were collected and screened based on the following criteria: (1) failure on attention check items (incorrect response to “Select option A”); (2) missing data; (3) patterned responding (consecutive selection of the same option). After excluding invalid responses, 2,016 valid questionnaires were retained for final analysis. The mean age of the students was 19.50 ± 1.57 years. Among them, 1,439 (71.4%) were male and 577 (28.6%) were female. This study was approved by the Academic Ethics Committee of Capital University of Physical Education and Sports in November 2025 (Approval No.: 2025A0153).

### 3.2 Research instruments

#### 3.2.1 Multidimensional Body-Self Relations Questionnaire (MBSRQ)

This study adopted the Chinese version of the Multidimensional Body-Self Relations Questionnaire, which was culturally adapted by Ma (2006). Two subscales were extracted: Health Evaluation and Comfort Evaluation, comprising 6 and 3 items, respectively, measured on a 5-point Likert scale. Health Evaluation refers to an individual’s perception of whether they are healthy or ill; higher scores indicate stronger health awareness. Comfort Evaluation refers to an individual’s affective experience regarding their own health; higher scores indicate that the individual perceives themselves as healthier and is more enthusiastic about participating in various activities to maintain or enhance their health. In this study, these were labeled Cognitive Body Assessment (CBA) and Affective Body Assessment (ABA), respectively. To examine the discriminant validity of CBA and ABA, this study employed confirmatory factor analysis (CFA) to compare the fit of a one-factor model versus a two-factor model. The results showed that the two-factor model exhibited better fit indices (CFI = 0.929, RMSEA = 0.018) than the one-factor model (CFI = 0.916, RMSEA = 0.037), indicating that although CBA and ABA are somewhat correlated, they are two distinct constructs. The Cronbach’s α coefficients for the two subscales were 0.713 and 0.673, respectively, indicating acceptable reliability.

#### 3.2.2 Sport Motivation Scale (SMS)

This study used a Chinese version of the Sport Motivation Scale, which was adapted and validated (Peng, 2012). Two subscales were selected: Intrinsic Motivation (IM) and Extrinsic Motivation (EM), each comprising 12 items measured on a 5-point Likert scale. Higher scores indicate higher levels of motivation. In this study, the Cronbach’s α coefficient for Intrinsic Motivation was 0.945, and for Extrinsic Motivation was 0.904. Only intrinsic and extrinsic motivation were included in the analysis.

#### 3.2.3 Physical Activity Rating Scale-3 (PARS-3)

The Physical Activity Rating Scale consists of three items (Liang, 1994), covering exercise intensity, exercise duration, and exercise frequency, each measured on a 5-point Likert scale. Physical activity (PA) volume was calculated as: physical activity volume = exercise intensity × (exercise duration-1) × exercise frequency. Higher scores indicate higher levels of physical activity. In this version, exercise intensity is rated on a scale of 1–5, with the highest level representing an intensity at or slightly below an individual’s ventilatory/lactate threshold rather than maximal exertion. The Cronbach’s α coefficient for this scale in this study was 0.700.

### 3.3 Statistical methods

Data collected were quantified and coded in Excel. Statistical analyses, including descriptive statistics, correlation analysis, and regression analysis, were conducted with SPSS 25.0 and MATLAB. Tests for mediation and moderated mediation were performed using PROCESS v4.0. Structural equation modeling was conducted with Stata 16.0. The significance level was set at α = 0.05.

## 4 Results and analysis

### 4.1 Common method bias

In this study, Harman’s one-factor test was conducted to assess the threat of common method bias. The results showed that unrotated exploratory factor analysis revealed six factors with eigenvalues > 1. The first factor accounted for 38.628% of the total variance, falling below the critical threshold of 40%, indicating that no single factor explained the majority of the variance. Thus, common method bias does not pose a serious problem in this dataset and does not constitute a major threat to the interpretation of the findings.

### 4.2 Current situation and correlation analysis of lifestyle behaviors and anxiety status of primary and secondary school students

As shown in Table 1, the Kolmogorov–Smirnov normality tests for BCE, BAE, IM, EM, and PA all showed statistical significance (*p* < 0.001, df = 2016), indicating that all variables were not normally distributed. Mann–Whitney U tests revealed no significant sex differences in BCE (*p* = 0.224 > 0.05) or BAE (*p* = 0.766 > 0.05). However, significant sex differences were found in both intrinsic and extrinsic motivation for exercise, as well as in PA (all *p* < 0.01). According to the conventional criteria for effect size (Cohen’s *d*), the effect sizes were approximately 0.2 for all three variables, which did not reach the 0.5 threshold, indicating small effects. Male students exhibited significantly higher exercise motivation and physical activity than female students.

**TABLE 1:** Sex differences across variables.

Variable	BCE	BAE	IM	EM	PA
Male (*n* = 1,439)	3.09 ± 0.61	3.10 ± 0.74	3.71 ± 0.73	3.53 ± 0.69	23.89 ± 21.48
Female (*n* = 577)	3.13 ± 0.63	3.09 ± 0.73	3.55 ± 0.77	3.40 ± 0.69	16.18 ± 18.78
*U*	400870.00	411686.00	367800.00	380323.00	305797.00
*P*	0.224	0.766	<0.001	0.003	<0.001
Cohen’s *d*	-0.0645077	0.0136051	0.2132575	0.1884058	0.382152

**FIGURE 1:**
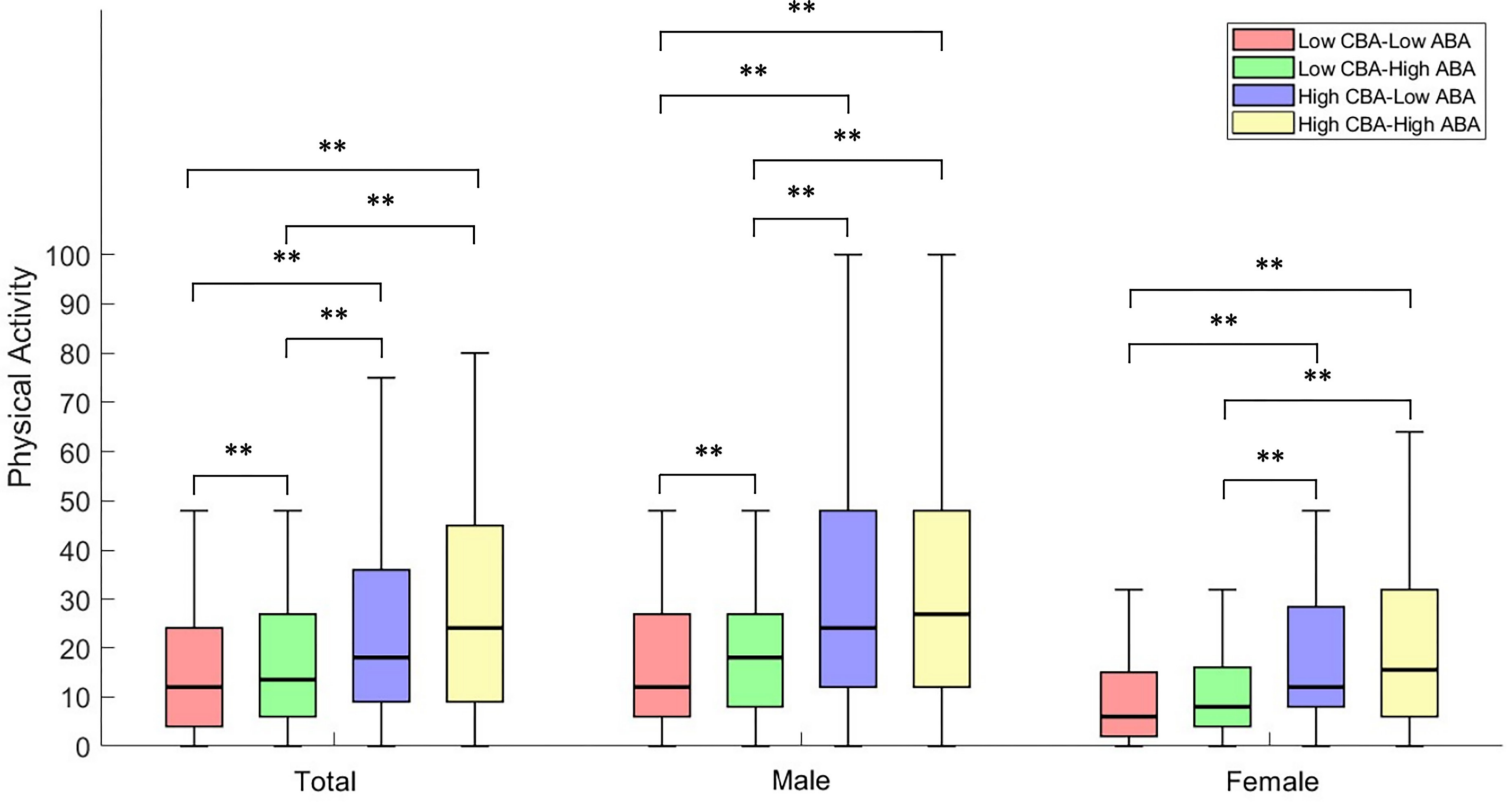
Group differences in PA across CBA and ABA categories. *P < 0.05, **P < 0.01, ***P < 0.001.

Following the standardized analysis procedure for Likert scales, this study used the theoretical midpoint of 3 as the cutoff. Respondents with mean scores ≥ 3 on CBA and ABA were classified into the high-cognition and high-affect groups, respectively, while those with mean scores < 3 were classified into the low-cognition and low-affect groups. The dichotomized CBA and ABA variables were then cross-combined to form four analytical groups: low cognition-low affect (*n* = 807), low cognition-high affect (*n* = 360), high cognition-low affect (*n* = 247), and high cognition-high affect (*n* = 602). A nonparametric Kruskal–Wallis H test was conducted, and the results showed significant differences in physical activity among the four groups (*H* = 136.185, *p* < 0.001), as shown in Figure 1. Further pairwise comparisons using Mann–Whitney U tests revealed that for the total sample, all pairwise comparisons showed significant differences (*p* < 0.01) except between the high cognition-low affect and high cognition-high-affect groups (*p* = 0.337 > 0.05). For the male subsample, all pairwise comparisons showed significant differences (*p* < 0.01) except between the high cognition-low affect and high cognition-high affect groups (*p* = 0.158 > 0.05). For the female subsample, significant differences (*p* < 0.01) were found only between low cognition-low affect and high cognition-low affect, between low cognition-low affect and high cognition-high affect, and between low cognition-high affect and high cognition-low affect, as well as between low cognition-high affect and high cognition-high affect. To control for Type I error inflation due to multiple comparisons, the Holm–Bonferroni method was applied to adjust *p*-values. With the exception of the high cognition-low affect vs. High cognition-high affect comparison, all other comparisons remained very robust and significant even after rigorous multiple comparison correction.

As shown in Table 2, Person correlation analysis revealed that cognitive body assessment was significantly positively correlated with intrinsic motivation (*r* = 0.260, *p* < 0.01), extrinsic motivation (*r* = 0.309, *p* < 0.01), and physical activity (*r* = 0.228, *p* < 0.01). Affective body assessment was also significantly positively correlated with intrinsic motivation (*r* = 0.367, *p* < 0.01), extrinsic motivation (*r* = 0.390, *p* < 0.01), and physical activity (*r* = 0.323, *p* <0.01). Given that all pairwise correlations were significant, further analyses could proceed.

**TABLE 2:** Correlation analysis matrix.

Variable	Intrinsic motivation	Extrinsic motivation	Exercise intensity	Exercise duration	Exercise frequency	PA	ABA	CBA
Intrinsic motivation	1	1	1	1	1	1	1	1
Extrinsic motivation	0.862**							
Exercise intensity	0.356**	0.317**						
Exercise duration	0.402**	0.348**	0.523**					
Exercise frequency	0.355**	0.363**	0.395**	0.396**				
PA	0.410**	0.395**	0.758**	0.724**	0.675**			
ABA	0.367**	0.390**	0.273**	0.233**	0.244**	0.323**		
CBA	0.260**	0.309**	0.184**	0.182**	0.204**	0.228**	0.615**	

*P < 0.05, **P < 0.01, ***P < 0.001.

**FIGURE 2:**
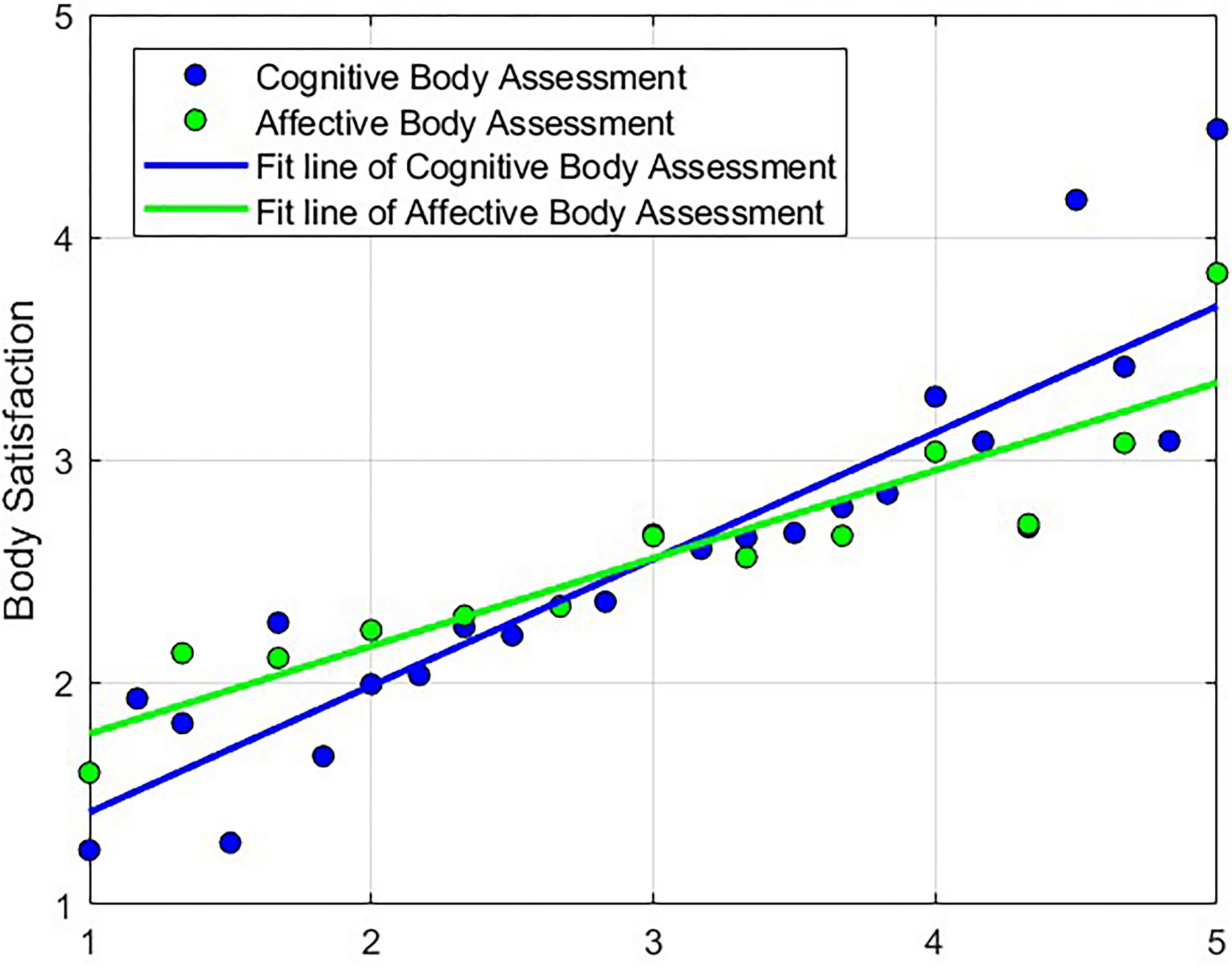
Scatter plots showing the relationship between cognitive body assessment and physical activity and affective body assessment and physical activity.

As shown in Figure 2, scatter plots were constructed for CBA and ABA, and PA. In the sample, both CBA and ABA exhibited positive correlations with PA. That is, individuals with higher levels of CBA tended to have higher levels of physical activity. Similarly, individuals with more positive ABA experiences exhibited higher levels of physical activity. However, the slopes of the two fitted lines differed noticeably. The fitted line for CBA was steeper, suggesting that CBA had a greater effect on physical activity than ABA, serving as a stronger predictor of higher physical activity levels. In terms of data distribution, the CBA data points were more dispersed, with notable outliers at the lower end, indicating greater individual variability. In contrast, the ABA data points were more concentrated, showed less dispersion, and demonstrated a more stable and consistent relationship with physical activity.

### 4.3 Mediation analysis

Using Model 4 developed by Hayes and a bootstrap estimation method, random resampling with replacement was performed on the sample of 2,016 college students to estimate the 95% confidence intervals (CIs) for the mediation effects. The results are presented in Table 3. For the pathway through intrinsic motivation, the indirect effect of cognitive body assessment on physical activity was 2.444, with a 95% CI of 1.744∼3.257, accounting for 31.37% of the total effect. For the pathway through extrinsic motivation, the indirect effect of cognitive body assessment on physical activity was 1.286, with a 95% CI of 0.443∼2.208, accounting for 16.51% of the total effect.

**TABLE 3:** Results of mediation analysis.

Path	Effect	Boot SE	LLCI	ULCI	Proportion
CBA→IM→PA	2.444	0.385	1.744	3.257	31.37%
CBA→EM→PA	1.286	0.449	0.443	2.208	16.51%
Direct effect	4.06	0.723	2.641	5.478	52.12%
Total effect	7.79	0.743	6.333	9.247	
ABA→IM→PA	2.618	0.412	1.857	3.461	28.25%
ABA→EM→PA	1.189	0.450	0.331	2.106	12.83%
Direct effect	5.460	0.620	4.244	6.677	58.92%
Total effect	9.267	0.605	8.082	10.454	

For the pathway through intrinsic motivation, the indirect effect of affective body assessment on physical activity was 2.618, with a 95% CI of 1.857∼3.461, accounting for 28.25% of the total effect. For the pathway through extrinsic motivation, the indirect effect of affective body assessment on physical activity was 1.189, with a 95% CI of 0.331∼2.106, accounting for 12.83% of the total effect. The 95% CIs for all pathways did not contain zero, indicating that both intrinsic motivation and extrinsic motivation significantly mediated the effects of cognitive body assessment and affective body assessment on physical activity.

### 4.4 Moderation analysis

Stepwise regression analysis was used to test the interaction between CBA and ABA. As shown in Table 4, in Models 1–3, with intrinsic motivation as the dependent variable, the R^2^ increased from 0.067 in Model 1 (with CBA as the sole independent variable) to 0.136 in Model 2 (after adding ABA), and further to 0.140 in Model 3 (after adding the interaction term CBA × ABA). The increase in model explanatory power was significant, and the coefficient for the interaction term was statistically significant (β = 0.061, *p* < 0.01), indicating that the synergistic effect between CBA and ABA enhanced their ability to predict intrinsic motivation. In Models 4–6, with extrinsic motivation as the dependent variable, the R^2^ increased from 0.096 in Model 4 (with CBA as the sole independent variable) to 0.160 in Model 5 (after adding ABA), and further to 0.161 in Model 6 (after adding CBA × ABA). The increase in model explanatory power was significant, and the coefficient for the interaction term was statistically significant (β = 0.040, *p* < 0.05), indicating that the synergistic effect between CBA and ABA enhanced their ability to predict extrinsic motivation.

**TABLE 4:** Moderation effects of affective body assessment on the relationship between cognitive body assessment and exercise motivation.

Variable	Intrinsic motivation			Extrinsic motivation		
	Model 1	Model 2	Model 3	Model 4	Model 5	Model 6
Constant	2.684**	2.406**	1.822**	2.416**	2.169**	1.788**
CBA	0.316**	0.067*	0.258**	0.347**	0.125**	0.250**
ABA		0.339**	0.532**		0.301**	0.427**
CBA*ABA			0.061**			0.040*
*R* ^2^	0.067	0.136	0.140	0.096	0.160	0.161
*ΔR* ^2^	0.067	0.069	0.003	0.096	0.064	0.002
*F*	145.640**	158.905**	108.980**	212.891**	191.169**	129.013**

*P < 0.05, **P < 0.01, ***P < 0.001.

**FIGURE 3:**
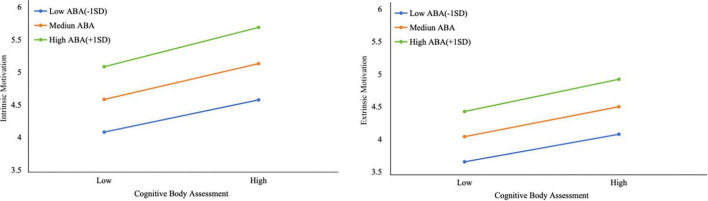
Simple slope plots of ABA moderating the effects of CBA on intrinsic motivation (left panel) and extrinsic motivation (right panel).

To further characterize the nature of the interaction, simple slope analyses were conducted to examine the effects of CBA on intrinsic and extrinsic motivation at low (mean-1 SD), medium (mean), and high (mean+1 SD) levels of ABA. As shown in Figure 3, for intrinsic motivation (left panel), the effect of CBA was significant at low (β = 0.494, *p* < 0.01), medium (β = 0.549, *p* < 0.01), and high (β = 0.604, *p* < 0.01) levels of ABA, with the effect increasing as ABA levels increased. For extrinsic motivation (right panel), the effect of CBA was also significant at low (β = 0.423, *p* < 0.01), medium (β = 0.459, *p* < 0.01), and high (β = 0.495, *p* < 0.01) levels of ABA, with the effect similarly increasing as ABA levels increased. These results indicate that the effects of CBA on both intrinsic and extrinsic motivation were enhanced with increasing levels of ABA, suggesting that ABA moderated the strength of the relationship between CBA and exercise motivation.

### 4.5 Moderated mediation analysis

This study used a moderated mediation model (Model 7 developed by Hayes) to test whether the level of ABA moderated the mediating role of intrinsic motivation and extrinsic motivation in the relationship between CBA and PA. To enhance estimation robustness, all continuous predictor variables were mean-centered prior to analysis to reduce the potential impact of multicollinearity on the estimation of interaction terms. Bootstrap resampling was performed 5,000 times, and the significance and stability of the moderated mediation effects were statistically inferred using bias-corrected 95% CIs. As shown in Table 5, for the CBA→IM→PA pathway, when ABA was at a low level, the indirect effect of intrinsic motivation was 0.350. As the level of ABA increased, the indirect effect values increased sequentially to 0.784 and 1.218. The 95% CIs for all conditions did not include zero. For the CBA→EM→PA pathway, when ABA was at a low level, the indirect effect of extrinsic motivation was 1.133. As the ABA level increased, the indirect effect values increased sequentially to 1.426 and 1.719. The 95% CIs for all conditions also did not include zero. These mediated pathways remained statistically significant across the entire observed range, indicating that ABA amplified the effect of CBA on PA through exercise motivation.

**TABLE 5:** Conditional indirect effects of CBA on PA through IM and EM at different levels of ABA.

Path	Level of ABA	Effect	Boot SE	LLCI	ULCI
HC→IM→PA	Low (M - 1 SD)	0.350	0.105	0.144	0.556
	Moderate (M)	0.784	0.385	0.140	1.503
	High (M + 1 SD)	1.218	0.503	0.170	2.150
HC→EM→PA	Low (M - 1 SD)	1.133	0.406	0.336	1.933
	Moderate (M)	1.426	0.366	0.712	2.156
	High (M + 1 SD)	1.719	0.473	0.766	2.645

### 4.6 Structural equation model

**FIGURE 4:**
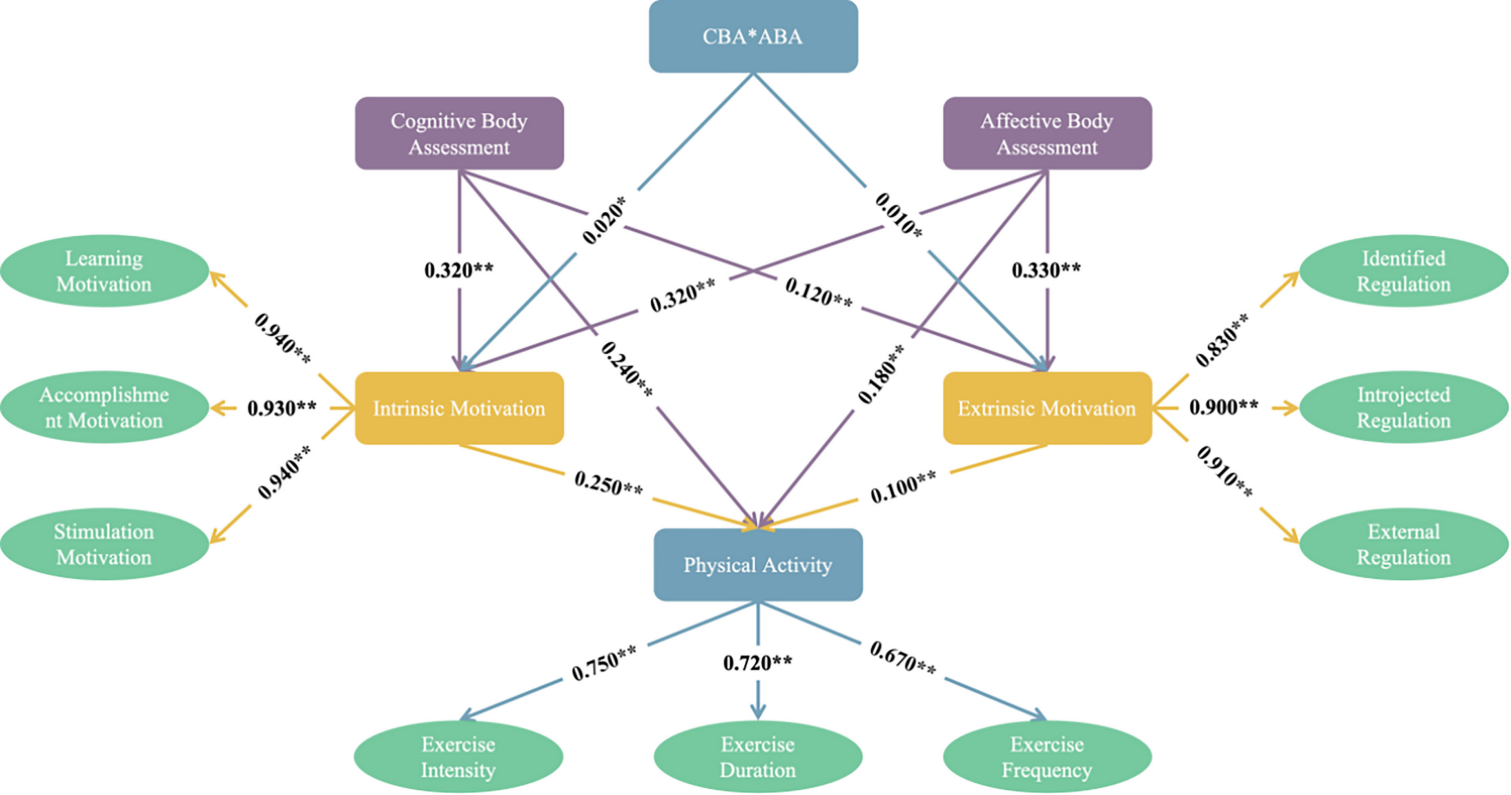
Structural equation model diagram. *P < 0.05, **P < 0.01, ***P < 0.001.

A structural equation model was constructed using Stata 16.0 to examine the relationships among health cognition, health affect, exercise motivation, and physical activity. The influence pathways were analyzed, and the results are shown in Figure 4. Because the chi-square value increases with sample size, which may lead to the rejection of any model, this study did not rely on the chi-square statistic as the sole criterion for model fit (Wen et al., 2004). As shown in the figure, health cognition had a more pronounced effect on intrinsic motivation (β = 0.320, *p* < 0.01), whereas health affect had a more pronounced effect on extrinsic motivation (β = 0.330, *p* < 0.01). Intrinsic motivation (β = 0.250, *p* < 0.01) had a larger effect on physical activity than extrinsic motivation (β = 0.100, *p* < 0.01). Parameter estimation results showed that the absolute values of the critical ratios for all path coefficients exceeded 1.96, and the standard errors were all below 1, indicating that the parameter estimates were not only statistically significant (*p* < 0.05) but also exhibited high estimation precision. In terms of global model fit, the root mean square error of approximation was 0.073, below the threshold of 0.08. The comparative fit index was 0.910, and the Tucker-Lewis Index was 0.904, both exceeding the generally accepted cutoff of 0.90, indicating good model fit.

## 5 Discussion

By conducting cross-group analyses combining cognitive body assessment and affective body assessment, this study found that groups with different cognition-affect combinations differed significantly in physical activity, indicating that individuals’ exercise behaviors are associated with the combination of cognitive and affective factors. Among participants in the high-cognition group, there was no significant difference in physical activity between those with high and low levels of affective evaluation. This finding indicates that among participants with high cognitive evaluation, affective level alone showed no significant association with physical activity, regardless of whether they had established a well-developed knowledge system regarding health. On the surface, this finding appears to diverge from the conventional assumption of cognition-affect synergy. Several interpretations may help account for this unexpected pattern. It is possible that, among individuals who have developed a sufficiently comprehensive knowledge base regarding health, cognitive evaluation alone may show a stronger statistical association with behavioral intentions, whereas the additional association of affective experience may diminish at higher cognitive levels, while the additional contribution of affective experience diminishes marginally at higher cognitive levels—suggesting that cognition may, to some extent, account for variance in behavioral intentions that is typically associated with affect in other contexts. However, this account remains speculative and warrants further investigation. Alternative explanations should also be considered. For instance, the measurement approach adopted in this study—where both CBA and ABA were derived from adjacent subscales of the same questionnaire—may have constrained within-group variance among participants with high cognitive scores, which could in turn be associated with reduced capacity to detect incremental effects of affective evaluation Future studies employing more differentiated measurement instruments or experimental designs are needed to further clarify the nature of cognition-affect interactions across varying levels of cognitive appraisal. Furthermore, there were no significant sex differences in either cognitive body assessment or affective body assessment, indicating that there is no pronounced sex gap in the cognitive and affective assessments of college students. However, significant sex differences were found in exercise motivation and physical activity. The mediation and pathway analyses revealed that the overall pathways from cognitive and affective assessments to exercise motivation and physical activity were valid; that is, the statistical pathways linking cognition and affect to motivation and behavior showed different patterns between female and male students. This difference may be better explained by internalized psycho-social-contextual factors than by the assessments themselves, such as gendered role expectations, body image anxiety, and sense of temporal control (Wilhelm et al., 2024; Ginis et al., 2007). Among college students with relatively similar levels of explicit cognitive and affective assessments, implicit psychological mechanisms related to motivation emerged as notable correlates of behavioral differences. Across sexes, the cognition–affect combination exhibited distinct differential patterns in its association with physical activity. Among male participants, the pattern of response was relatively straightforward, with their physical activity showed a stronger association with the combination of cognition and affect. Among female participants, the observed pattern of associations was more complex. The results showed that within the same level of cognition, differences in affective evaluation were not significantly associated with differences in physical activity. In contrast, differences in cognition were consistently associated with significant differences in physical activity One possible interpretation is that most female students may not have established sufficiently strong or effective affective associations with physical activity. It is also possible that they tend to face greater internal and external barriers when participating in exercise, may be more susceptible to body image concerns such as weight management, and might exhibit lower motivation and sustainability for long-term exercise maintenance (Elmahgoub et al., 2025). In addition, the female subsample (*N* = 577) was substantially smaller than the male subsample (*N* = 1,439) in this study, which may have resulted in insufficient statistical power to detect certain between-group differences. Furthermore, the PARS-3 may not be sufficiently sensitive to capture physical activity types commonly engaged in by female students, such as yoga, dance, and other light-to-moderate intensity activities. Future research should employ larger female samples and more comprehensive physical activity assessment tools to further validate the observed sex difference patterns.

Building on the identification of cognition–affect combinations and sex differences, the mediation and moderation analyses further revealed the psychological dynamics underlying physical activity. Both intrinsic and extrinsic motivations were significant mediators in the statistical pathways from cognitive and affective assessments to physical activity, shedding light on a hierarchical psychological dynamic model of physical activity. Intrinsic motivation emerged as a key motivational factor, with its mediating effect size (28.25–31.37%) being approximately twice that of extrinsic motivation (12.83–16.51%), further supporting the core tenet of self-determination theory that intrinsic drive, which originates from interest and satisfaction, provides a more solid foundation for sustained behavior. In the current sample, participation driven by interest and autonomous choice (rather than external pressure) showed a statistical association with higher levels of behavioral sustainability. In addition, college students are situated at a critical period of autonomy development, and their tendency to prioritize “choice” over “obligation” may further reinforce the relative predominance of intrinsic motivation. Nonetheless, the significant mediating role of extrinsic motivation—though comparatively modest—merits attention. This finding implies that external incentives (e.g., academic credits, rewards) may still function as effective catalysts in the initial phase of behavioral change, with the essential challenge being their gradual internalization into autonomous forms of motivation. This finding elevates the static description of cognition-affect two-dimensional groups into a dynamic process spanning from cognition and affect through motivation to behavior, clarifying the critical transmission role of exercise motivation. Affective assessment was not only a parallel correlate alongside cognitive evaluation but also a significant moderator in the relationship linking health cognition to exercise motivation. The moderation and moderated mediation analyses indicated that the level of affective assessment significantly moderated the strength of the relationship between health cognition and exercise motivation-particularly intrinsic motivation-and amplified the indirect effect of health cognition on physical activity through exercise motivation. Positive affective assessment appeared not only as an independent correlate but also as a factor that may be associated with a stronger statistical association between cognition and physical activity. When individuals hold more positive affective assessments, their cognitive assessments tend to correspond with stronger identification and perceived value, which in turn are associated with higher motivation for exercise. Combined with the finding from the group analysis that there were significant differences in physical activity between the low cognition-low-affect and low cognition-high affect groups, this indicates that among individuals with lower cognitive levels, positive affective assessment may show a compensatory statistical pattern in relation to physical activity However, combined with the finding of no significant difference in physical activity between the high cognition-high-affect and high cognition-low affect groups, this suggests that once individuals’ cognitive assessment levels have increased, the association between cognition and motivation may appear more consistent at higher cognitive levels, such that variations in affective assessment alone show little additional association with behavioral differences.

Promoting physical activity among college students is a systematic endeavor. A comprehensive strategy should be implemented based on the dynamic “Cognition-Affect-Motivation-Behavior” model, integrating individual psychological empowerment, optimization of supportive environments, and the establishment of sustainable mechanisms. The core lies in stimulating intrinsic motivation through the synergistic intervention of cognition and affect, lowering the threshold for participation by creating a supportive environment, and, through precisely tailored guidance and ongoing support, helping college students transform exercise from a task into an enjoyable, autonomous, and sustainable lifestyle habit. First, a dual-track educational approach targeting both health cognition and health affect should be implemented, enabling them to work synergistically to spark the endogenous motivation for individuals to engage in exercise. Experiential courses can promote the internalization of health knowledge and foster positive affective associations, facilitating the transformation of exercise behavior from “what should be done” to “what is actually done.” Offering a variety of exercise options that allow for independent choice and perceived value identification can enhance students’ autonomy and sense of accomplishment. Taking sex differences into account, for female students, strategies should emphasize the reshaping of body image, the creation of supportive environments, and the translation of health cognition into action. For male students, given their greater sensitivity to the cognition-affect combination, motivation can be enhanced through competitive activities and role identity reinforcement. Second, supportive ecosystems should be created to lower barriers to participation. By enhancing campus sports facilities and services and fostering a convenient and diverse exercise environment, and by combining a vibrant campus sports culture encompassing a variety of events, community activities, and social media campaigns, regular physical activity can be integrated into credit systems, daily routines, and classroom transitions. Lowering the threshold to participation is key to making exercise an integral part of campus life. Finally, long-term mechanisms should be established to transition from short-term interventions to sustained habits. Precise strategies should be implemented for groups with different cognition-affect combinations. For the low-cognition or high-affect group, participation can be encouraged by initiating appealing and socially oriented activities, thereby promoting cognition through affectation. For the high-cognition group, skill refinement and content innovation can help individuals break through plateaus. Digital tools can be used for dynamic monitoring and feedback. A multidisciplinary, integrated promotion system involving athletics, student affairs, and psychological counseling should be established to encompass education, services, and support. The ultimate goal is to transform exercise from a short-term intervention into a lifelong habitual practice.

## 6 Conclusion

Through empirical analysis, this study enriches the body of empirical research on the “cognition-affect” dual-system theory in exercise psychology as applied to the college student population, clarifies the mechanisms of action of the key variables, and reveals the synergistic influence of cognitive body assessment and affective body assessment on college students’ physical activity. At low cognitive levels, positive affect can compensate for cognitive deficits; at high cognitive levels, merely enhancing affect may not be sufficient to increase physical activity. Exercise motivation significantly mediates the relationship between cognitive assessment, affective assessment and physical activity, with the mediation effect of intrinsic motivation being significantly stronger than that of extrinsic motivation, thus clarifying the dynamic pathway of “Cognition-Affect-Motivation-Behavior.” Affective body assessment also functions as a moderator, amplifying the indirect effect of cognitive assessment on physical activity through motivation. Additionally, no significant sex differences were found in either cognitive or affective body assessment, but sex differences were observed in exercise motivation and physical activity. Males showed greater sensitivity to the cognition-affect combination, whereas females were more influenced by body image anxiety and other factors, with cognition exerting a more pronounced effect on physical activity. Based on the dynamic “Cognition-Affect-Motivation-Behavior” model, a comprehensive strategy should be implemented that integrates dual-track education targeting both cognition and affect, the creation of supportive campus environments, and the construction of long-term mechanisms. Precise interventions tailored to sex and cognition-affect combinations should be developed to help college students internalize exercise as a sustainable lifestyle habit.

## 8 Research limitations and outlook

Although this study systematically examined the relationships and mechanisms involving cognitive body assessment, affective body assessment, exercise motivation, and physical activity among college students, several limitations should be noted. First, this study employed a cross-sectional design, which could not accurately establish causal relationships among the variables. Future research could further investigate these causal pathways by adopting longitudinal or experimental designs, and incorporate objective measurement methods such as accelerometers to more accurately assess the intensity and frequency of physical activity. Second, the measurement of core variables relied on self-report methods, which may be subject to self-report biases and may not fully capture the objective levels of individuals’ authentic cognitive, affective, or physical activity statuses. Third, the study sample did not fully account for the heterogeneity within the college student population, and it was predominantly male (71.4%), with females accounting for only 28.6%, both of which limit the generalizability of the findings. Future research could further refine sample classifications and conduct stratified analyses based on the characteristics of different subgroups of college students, thereby enhancing the specificity and generalizability of the conclusions.

## Data Availability

The original contributions presented in this study are included in this article/supplementary material, further inquiries can be directed to the corresponding author.
